# Extracting Social Determinants of Health from Inpatient Electronic Medical Records

**DOI:** 10.23889/ijpds.v9i5.2717

**Published:** 2024-09-18

**Authors:** Elliot Martin, Adam D'Souza, Vineet Saini, Karen Tang, Hude Quan, Cathy Eastwood

**Affiliations:** 1 Centre for Health Informatics, University of Calgary; 2 Provincial Research Data Services, Alberta Health Services; 3 Public Health Evidence and Innovation, Alberta Health Services; 4 Department of Community Health Science, University of Calgary; 5 Department of Medicine, University of Calgary

## Objective

Social determinants of health (SDOH) have been shown to be important predictors of health outcomes. Here we assess how best to extract SDOH variables from inpatient electronic medical record (EMR) data.

## Approach

Four social determinants were targeted: patient language barriers, employment status, education, and whether the patient lives alone. Inpatients aged 18 and older with records in the Calgary-wide EMR system were studied. Algorithms were developed on January 2019 hospital admissions (n=8,999), and validated on January 2018 hospital admissions (n=8,839). SDOH documented as structured data, which can be easily queried, were compared against those extracted from unstructured free-text notes.

## Results

More than twice as many patients had an unstructured note documenting a language barrier than in the structured data; 12% of patients indicated by notes to be living alone had a partner in their structured marital status. The Positive Predictive Value (PPV) of the elements extracted from notes was high, at 99% (95% CI 94.0%-100.0%) for language barriers, 98% (95% CI 92.6%-99.9%) for living alone, 96% (95% CI 89.8%-98.8%) for unemployment, and 88% (95% CI 80.0%-93.1%) for retirement.

## Conclusions

It is possible to extract SDOH elements from free text notes with high PPV. SDOH documentation was largely missing in structured data, and sometimes misleading.

## Implications

Free text notes can be a fruitful source of information for projects using SDOH variables, such as machine learning/AI or health services research, and can offer insights not available from the structured data elements.

